# Novel design and deployment of orthologous genic SSR markers in *Eucalyptus camaldulensis* Dehnh

**DOI:** 10.1186/1753-6561-5-S7-P51

**Published:** 2011-09-13

**Authors:** Kengavanar Nagabhushana, Prasad Suresh Hendre, Navin Sharma, Rajkumar Rathinavelu

**Affiliations:** 1 Plant Molecular Biology, ITC R&D Centre, Bangalore-58, India

## Background

*Eucalyptus camaldulensis* is a widely planted tree species in India, because of its rapid growth and adaptability to dry regions. Genetic improvement through informed breeding of *E. camaldulensis* largely depends on availability of molecular markers, linkage maps and genome information. Microsatellite markers, also called as simple sequence repeats (SSRs) have wide application due to their unique advantages over other marker systems. In Eucalyptus, SSR markers have been used in various breeding applications from DNA fingerprinting to QTL mapping [1,4]. Despite of their advantages, the major drawback is the time required for their development [6]. There are less than twenty five *E. camaldulensis* specific SSR markers available in the public database [5]. Although, there are large number of SSRs available in other *Eucalyptus* species [2,3] , their species transferability in *camaldulensis* is questionable for practical use. Nevertheless, the existing *camaldulensis* specific SSRs are insufficient for developing linkage maps, QTL and comparative mapping studies. Besides, EST based markers are handicapped due to exclusion of introns, which sometimes lead to compromise on product sizing. Therefore we have modeled a novel strategy that targets highly conserved domain in the genic region using both publicly available ESTs as well as genome sequences.

## Methods

### Targeting conserved genic region

**Figure 1:**
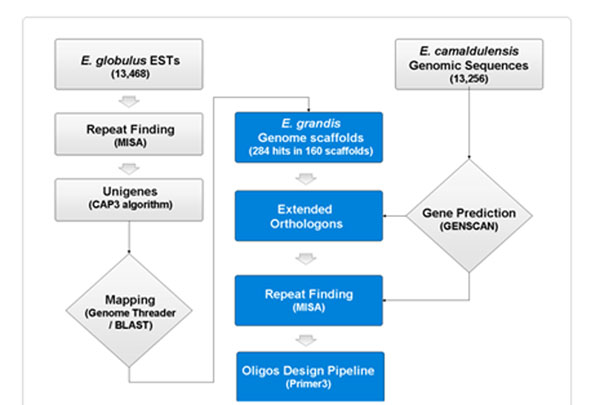
Strategy summarizing the in silico development of orthologus SSRs

A set of publicly available *E. globulus* ESTs were assembled as unigenes using CAP3 algorithm and mined for repeat motifs using MISA program [http://pgrc.ipk-gatersleben.de/misa/]. The repeat rich unigenes were marked and mapped on the whole genome scaffolds of *E. grandis.* The sequence of mapped co-ordinates were extracted from EUCAGEN database [http://web.up.ac.za/eucagen/] and further validated for repeat motifs and the presence of exons using MISA and GENSCAN (http://genes.mit.edu/genscan.html). A total of 300 ± base pairs were marked in the genic region of scaffolds flanking the repeat motifs and further primers were designed on the flanking region of repeat motifs using the Primer3 tool [http://primer3.sourceforge.net/], following the appropriate parameters (Figure 1). The entire pipeline was automated and multi-threaded using a set of in-house PERL programs.

### PCR, multiplexing and genotyping

We adopted an innovative two-tier polymerase chain reaction (PCR) system to reduce the cost 12 times on labeled oligo synthesis. The first PCR was performed with M13 tagged *forward* primer at 5’ end whereas the second PCR was performed with fluorescently labeled M13 as *forward* primer and SSR *reverse* primer. Multiplexing was carried out at post PCR stage. The first PCR consisted of template DNA (5 ng), primers (2 pM), 10X PCR buffer, dNTPs (1 µM), MgCl_2_ (1.5mM) and Amplitaq Gold *Taq* DNA polymerase (0.25 U). The PCR profile consisted of denaturing the template DNA at 94^o^C for 5 min, followed by 35 cycles, each at 94^o^C for 30 sec, 50-65^o^C for 30 sec and 72^o^C for 1 min, followed by 72^o^C for 8 min. The first PCR products were resolved on 2 % high resolution agarose. The second PCR consisted of template DNA (2 ng), 2 pM each of forward (labeled M13) and reverse (SSR) primer, 10X PCR buffer, dNTPs (1 µM), and 0.5U of *Taq* DNA polymerase. The PCR conditions remained same except reduced number of cycles to 20. The amplicons were resolved using ABI 3730 sequencer and each amplicon was manually validated for their allele size.

## Results and conclusion

**Figure 2:**
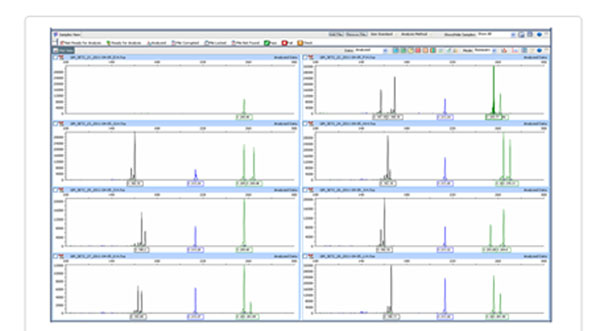
Electropherogram of multiplexed PCR products of primers IME3126 (black), IME3130 (blue) and IME3285 (green) in eight clones of *E. camaldulensis*

About 13,441 *E. globulus* ESTs and 13,380 *camaldulensis* EST/genomic sequences were collected from public database and mined for repeat motifs. A total of 2,330 repeat motifs were identified on 1873 ESTs of which, 1159 were di; 1128 tri and 43 were tetra repeats. There were more than 290 repeats found to be in compound form. The repeat containing ESTs were assembled to a total of 301 unigenes. These ESTs were mapped on *E. grandis* genome scaffolds. A total of 124 SSR positive scaffolds were identified and used for designing 230 primer pairs. Primers were standardized using gradient PCR at appropriate annealing temperatures. Of the 230 primers, 179 were successfully amplified and validated in *E. camaldulensis* resulting in 77.82 % success. About 95 % of the primers were amplified as single and clean product, indicating their locus specificity (Figure 2). These markers were validated in 4 *Eucalyptus* and 2 *Corymbia* species with multiple alleles ranging from 4 to 12. Surprisingly, 92 % of cross species amplification was observed within the genus Eucalyptus, while only 21% in *Corymbia*. Higher species transferability in *Eucalyptus* genus shows the power of design as they originate from conserved domain. Further to validate these markers in *E. camaldulensis*, some of the selected primers were successfully utilized for parentage analysis, confirmation of interspecific hybrid and genotyping of seedling seed orchard in *E. camaldulensis*. Unlike SSR markers developed from conventional *in silico* methods, orthologous SSRs resulted in very high success rate in *Eucalyptus* species due to targeted repeats in conserved domain. Our present strategy successfully demonstrated the power of orthologous SSR makers and its application in informed breeding in *E. camaldulensis*.
